# Positron Emission Tomography-Computed Tomography Parameters Predict Efficacy of Immunotherapy in Head and Neck Squamous Cell Carcinomas

**DOI:** 10.3389/fonc.2021.728040

**Published:** 2021-09-28

**Authors:** Songtao Zhang, Runfang Zhang, Wenbo Gong, Chao Wang, Chen Zeng, Yifei Zhai, Qigen Fang, Liyuan Dai

**Affiliations:** Department of Thyroid and Head Neck, Affiliated Cancer Hospital of Zhengzhou University, Henan Cancer Hospital, Zhengzhou, China

**Keywords:** PD-1 inhibitor, immunotherapy, head and neck squamous cell carcinoma, metabolic tumor volume, SUVmax

## Abstract

**Background:**

This study aims to assess the association between positron emission tomography-computed tomography (PET-CT) parameters and the response to immune checkpoint inhibitors in unresectable head and neck squamous cell carcinoma (HNSCC).

**Methods:**

A total of 105 patients receiving immunotherapy (pembrolizumab or sintilimab with/without cisplatin) were retrospectively enrolled in this study; pretreatment data regarding metabolic tumor volume (MTV) and maximum standardized uptake value (SUVmax) were collected. The primary interest of the study was objective response rate (ORR), and the secondary was progression−free survival (PFS).

**Results:**

The mean total MTV was 40.6 cm^3^ (range: 8.5–100.3), ORRs in tumors with total MTV of ≥40.6 and <40.6 cm^3^ were 43.1% and 23.1%, respectively; the difference was statistically significant (*p* = 0.018). Survival analysis indicated similar PFS rates in the two groups (*p* = 0.057). The mean total SUVmax was 12.5, ORRs in tumors with total SUVmax ≥12.5 and <12.5 were 40.0% and 26.0%, respectively; the difference was not significant (*p* = 0.092). Survival analysis reported patients with total SUVmax of ≥12.5 had significantly worse PFS (*p* = 0.001) than patients with total SUVmax of <12.5.

**Conclusions:**

In HNSCC, total MTV ≥40.6 cm^3^ translated into improved clinical response but not into better PFS; total SUVmax had no effect on clinical response, but total SUVmax ≥12.5 was associated with worse PFS.

## Introduction

Head and neck squamous cell carcinoma (HNSCC) is the seventh commonest malignancy in the world (1), even treated with multiple procedures, numerous patients develop locoregional recurrence and/or distant metastasis and can only receive palliative chemotherapy if there is no chance of salvage surgery (2). The emergence of immune checkpoint inhibitors, of which PD-1 inhibitor is the prototype, greatly reformed the treatment blueprint. Burtness et al. (3) analyzed the definite treatment role of pembrolizumab in 882 patients with untreated incurable HNSCC, and noted that in the combined positive score (CPS) of 20 or more patients, pembrolizumab alone improved overall survival (OS) compared with those on cetuximab with chemotherapy (14.9 *vs*. 10.7 months); in the CPS of one or more patients, pembrolizumab with chemotherapy achieved longer OS time than other regimen. Recently, the Food and Drug Administration (FDA) approved the use of PD-1 inhibitor (pembrolizumab) with or without cisplatin as the first-line therapy for recurrent or metastatic HNSCC (4).

However, the overall response rate to PD-1 inhibitor monotherapy is about 20%; thus, there is a need to identify biomarkers that will help with patient selection (5). Some researchers have analyzed the predictive value of PD-L1 expression (6), peripheral lymphocyte counts (7), total tumor burden (8), and gut microbiome (9) in PD-1-based immunotherapy efficacy, although this is still controversial.

PET-CT is widely used for detecting disease metastasis in clinical practice, and its parameters including maximum standard uptake value (SUVmax) and metabolic tumor volume (MTV), are significantly associated with patient survival in HNSCC (10, 11).

Therefore, this study aims to assess the association between PET-CT parameters and response to immune checkpoint inhibitors in unresectable HNSCC.

## Materials and Methods

### Ethical Consideration

Our hospital institutional research committee approved our study, and all participants signed an informed consent agreement. All methods were performed in accordance with the relevant guidelines and regulations. All procedures performed in studies involving human participants were in accordance with the ethical standards of the institutional and/or national research committee and the 1964 Helsinki Declaration and its later amendments or comparable ethical standards.

### Patient Selection

Between January 2018 and January 2021, medical records of HNSCC patients were retrospectively reviewed; inclusion criteria were as follows: those with disease defined as unresectable; those who received pembrolizumab or sintilimab with/without cisplatin (docetaxel 75 mg/m^2^ and cisplatin 100 mg/m^2^, on day 1; fluorouracil by continuous infusion 1,000 mg/m^2^, on days 1 to 5, TPF) as palliative treatment after multidisciplinary consultation, based on Chinese Society of Clinical Oncology guidelines for recurrent and metastatic HNSCC; those who had a PET-CT scan performed within 1 week prior to treatment. Patients were excluded if they had insufficient follow-up data or had received other treatment for the primary or recurrent disease. Information regarding demographic and pathologic data, Eastern Cooperative Oncology Group (ECOG) performance, PET-CT parameters, prior treatment, and follow-up was extracted and analyzed.

### Palliative Treatment

Pembrolizumab (Keytruda, Merck & Co, Kenilworth, NJ, USA) or sintilimab (Daboshu, Innovent Biologics, Suzhou, China) was used solely or concurrently with TPF chemotherapy based on comprehensive consideration of the patient’s economic status and willingness, health condition, PD-L1 expression, and so on for HNSCC patients from January 2018. Pembrolizumab or sintilimab was given intravenously at a dose of 200 mg every 3 weeks until intolerable toxicity or disease progression occurred. Six cycles of TPF chemotherapy was given.

### Immunohistochemical Analysis

From July 2013, immunohistochemical analysis of p16 (Wuhan Boster Biotechnology Co., Ltd., Wuhan, China) was performed for every patient with HNSCC. The level of positivity of p16 overexpression was consistent with that in previous studies (12): 0–+, defined as less than 25% tumor staining; ++, defined as 25%–50% tumor staining; +++, defined as 50%–75% tumor staining; and ++++, defined as more than 75% tumor staining. Tumors with +++ and ++++ were classified as having p16 positivity.

PD-L1 expression was assessed using the 22C3 pharmDx assay (DAKO, Glostrup, Denmark) and calculated by CPS, it was defined as the number of PD-L1-positive cells divided by the total number of tumor cells ×100; a minimum of 100 viable tumor cells must have been present for the specimen to be considered evaluable (3).

### Important Variable Definitions

A current smoker was defined as one who had smoked at least 10 cigarettes per day for at least 10 years, a former smoker was defined as one who had quit smoking for at least 2 years prior to this study, a never smoker was defined as one who had smoked no more than 100 cigarettes in his lifetime (13). Total SUVmax was calculated as the sum of the SUVmax of all measurable lesions by RECIST, version 1 (14). Total MTV was calculated as the sum of the MTV of all measurable lesions by RECIST, version 1 (14).

### PET-CT Scheme

PET-CT (GE Healthcare, Milwaukee, WI, USA) was performed by several scanners. Before the scan, patients were required to fast for at least 6 h. If glucose levels were >200 mg/dl, PET-CT scan was postponed. Every patient received 10–20 mCi of [^18^F] FDG dosed according to his or her weight. Axial PET and diagnostic CT images were obtained from the calvarial vertex through the upper thighs after urinary voiding. Emission images were obtained after radiopharmaceutical injection, 60 min later. During the CT scan, there was no contrast medium used. The images were reconstructed to the thickness of a 2.5-mm slice. The SUVmax was measured for all suspicious lesions. For every suspicious lesion, the isocontour region of interest centered on the maximum value pixel was drawn automatically with workstation tools generating the SUV max of the region. A SUVmax cutoff of 2.5 MBq/g was used to indicate malignancy.

### Follow-Up Protocol

The patient was usually examined by palpation and image assessment after every two to four cycles of treatment; timely evaluation was performed if there was any new sign of disease progression or other complications.

### Study Endpoint

The primary study interest was objective response rate (ORR); the secondary interest included progression−free survival (PFS) and OS. Responses were formulated as complete response (CR), partial response (PR), stable disease (SD), and progressive disease (PD) according to RECIST, version 1 (14), and were assessed every 8 weeks, with the overall treatment response defined as the best response recorded from the initial treatment to disease progression or death. The ORR referred to the proportion of patients achieving CR or PR. PFS was calculated as the time from the date of diagnosis to documented disease progression, or death from any cause. OS was calculated as the time from the date of diagnosis until death from any cause (3).

### Statistical Analysis

The Chi-square test (univariate analysis) was used to analyze the association between clinicopathologic variables, PET-CT parameters, and ORR, and the factors which were significant in univariate analyses were then analyzed in multivariate analysis to detect the independent predictors. The Kaplan-Meier method was used to compare the PFS and OS. Cox model was used to determine the independent prognostic factors. All statistical analyses were performed using SPSS 20.0, and a *p* < 0.05 was considered to be significant.

## Results

### Baseline Data

In the 105 patients, 75 (71.4%) were male and 30 (28.6%) were female with a mean age of 56.8 years (range: 38–79). Twenty-nine (27.6%) of them had an ECOG score of 0, 61 (58.1%) had a score of 1, and 15 (14.3%) had a score of 2. Twenty (19.0%) patients were current smokers, 61 (58.1%) were former smokers, and 24 (22.9%) were never smokers. Thirty-five (33.3%) patients had a primary disease, 70 (66.7%) had recurrent/metastatic disease, and had all received prior treatment in form of surgery or radiotherapy; 35 of them had also received chemotherapy. Primary tumors were located in the oral cavity in 20 (19.0%) patients, in the oropharynx in 31 (29.5%), in the larynx in 32 (30.5%), and in the hypopharynx in 22 (21.0%). Positivity of p16 occurred in 11 (10.5%) patients. The mean total SUVmax was 12.5 (range: 4.7–48.6), while the mean total MTV was 40.6 cm^3^ (range: 8.5–100.3). Fourteen (13.3%) patients had a CPS of less than 1, 66 (62.9%) had a CPS greater than 1 but less than 20, and 25 (23.8%) had a CPS no less than 20. Forty-six (43.8%) patients received immunotherapy alone, and 59 (56.2%) received both immunotherapy and TPF chemotherapy (**Table 1**).

**Table 1:** Baseline data of the enrolled 105 patients.

Parameter	*N* (%)
Age	
<40	3 (2.9%)
≥40	102 (97.1%)
Sex	
Male	75 (71.4%)
Female	30 (28.6%)
Eastern Cooperative Oncology Group score	
0	29 (27.6%)
1	61 (58.1%)
2	15 (14.3%)
Smoker	
Current	20 (19.0%)
Former	61 (58.1%)
Never	24 (22.9%)
Disease classification	
Primary	35 (33.3%)
Recurrent/metastatic	70 (66.7%)
Prior treatment	
None	35 (33.3%)
Surgery, radiotherapy	35 (33.3%)
Surgery, chemoradiotherapy	35 (33.3%)
Primary tumor site	
Oral cavity	20 (19.0%)
Oropharynx	31 (29.5%)
Larynx	32 (30.5%)
Hypopharynx	22 (21.0%)
Positivity of p16	11 (10.5%)
Total SUVmax	
<12.5	50 (47.6%)
≥12.5	55 (52.4%)
Total MTV (cm^3^)	
<40.6	47 (44.8%)
≥40.6	58 (55.2%)
Combined positive score	
<1	14 (13.3%)
1–20	66 (62.9%)
≥20	25 (23.8%)
Palliative treatment	
PD-1 inhibitor	46 (43.8%)
PD-1 inhibitor + TPF	59 (56.2%)

SUVmax, max standard uptake value; MTV, metabolic tumor volume; TPF, docetaxel + cisplatin + fluorouracil.

### Response to Treatment and Its Potential Predictors

A total of 35 patients showed positive clinical response; there were four cases of CR and 31 cases of PR, the overall ORR was 33.3%, and SD and PD occurred in 15 (14.3%) and 55 (52.4%) patients, respectively.

Tumors with p16 positivity had an ORR of 72.7%, which was significantly higher than 28.7% in tumors without p16 positivity (*p* = 0.006). The ORRs in tumors with total MTV ≥40.6 cm^3^ and<40.6 cm^3^ were 43.1% and 23.1%, respectively; the difference was significant (*p* = 0.018). In tumors with CPS ≥20, the ORR was 52.0%, it was apparently higher than those of the other two subgroups (*p* = 0.044). Compared with immunotherapy alone, the addition of TPF yielded a significantly increased ORR of 42.4% (*p* = 0.026). The ORRs in tumors with total SUVmax ≥12.5 and<12.5 were 40.0% and 26.0%, respectively; the difference was not significant (*p* = 0.092). Moreover, the treatment response had no association with age, sex, ECOG score, or smoking status (all *p* > 0.05) (**Table 2**). In further multivariate analysis, the factor total MTV remained an independent predictor of response (**Table 3**).

**Table 2:** Univariate analysis of the association between general variables and treatment response in the 105 patients.

Variables	Response (CR/PR)	Nonresponse (SD/PD)	*p*-Value
	*N* = 35	*N* = 70	
Age			
<40	0	3 (100%)	
≥40	35 (34.3%)	67 (65.7%)	0.549
Sex			
Male	23 (30.7%)	52 (69.3%)	
Female	12 (40.0%)	18 (60.0%)	0.359
ECOG^#^			
0	9 (31.0%)	20 (69.0%)	
1	22 (36.1%)	39 (63.9%)	
2	4 (26.7%)	11 (73.3%)	0.751
Smoker			
Current	7 (35.0%)	13 (65.0%)	
Former	20 (32.8%)	41 (67.2%)	
Never	8 (33.3%)	16 (67.7%)	0.984
Disease classification			
Primary	13 (37.1%)	22 (62.9%)	
Recurrent/metastatic	22 (31.4%)	48 (68.6%)	0.693
Prior treatment			
None	13 (37.1%)	22 (62.9%)	
Surgery + radiotherapy	11 (31.4%)	24 (68.6%)	
Surgery + chemoradiotherapy	11 (31.4%)	24 (68.6%)	0.842
Primary tumor site			
Oral cavity	7 (35.0%)	13 (65.0%)	
Oropharynx	13 (41.9%)	18 (58.1%)	
Larynx	10 (31.3%)	22 (68.7%)	
Hypopharynx	5 (22.7%)	17 (77.3%)	0.525
p16			
Positive	8 (72.7%)	3 (27.3%)	
Negative	27 (28.7%)	67 (71.3%)	0.006
Total SUVmax			
<12.5	13 (26.0%)	37 (74.0%)	
≥12.5	22 (40.0%)	33 (60.0%)	0.092
Total MTV (cm^3^)			
<40.6	10 (21.3%)	37 (78.7%)	
≥40.6	25 (43.1%)	33 (56.9%)	0.018
Combined positive score			
<1	2 (14.3%)	12 (85.7%)	
1–20	20 (30.3%)	46 (69.7%)	
≥20	13 (52.0%)	12 (48.0%)	0.044
Drug combination			
None	10 (21.7%)	36 (78.3%)	
TPF	25 (42.4%)	34 (57.6%)	0.026

ECOG, Eastern Cooperative Oncology Group score; SUVmax, max standard uptake value; MTV, metabolic tumor volume; TPF, docetaxel + cisplatin + fluorouracil; CR/PR, complete response/partial response; SD/PD, stable disease/progressive disease.

**Table 3:** Multivariate analysis of the association between general variables and treatment response in the 105 patients.

Variable	Predictor for response	
	*p*-Value	OR [95% CI]
p16 (positive *vs*. negative)	0.356	2.16 [0.762–6.448]
Total MTV (≥40.6 *vs*. <40.6)	0.004	4.326 [1.327–8.332]
Combined positive score		
<1		
1–20	0.032	2.198 [1.032–5.432]
≥20	0.002	6.438 [1.983–15.725]
Drug combination (TPF *vs*. none)	0.017	3.218 [1.836–7.338]

MTV, metabolic tumor volume; TPF, docetaxel + cisplatin + fluorouracil.

### Toxicity to Treatment

Adverse events occurred in all patients, with 268 grades 1–2 events and 12 grades 3–4 events. The most common grades 1–2 and grades 3–4 events were anorexia and neutropenia, respectively. Two patients died of serious adverse reaction (**Table 4**).

**Table 4:** Adverse events in the 105 patients.

Event	Number (%)	
	Grades 1–2	Grades 3–4
Anorexia	57 (54.3%)	
Nausea	43 (41.0%)	
Fatigue	40 (38.1%)	
Constipation	35 (33.3%)	
Stomatitis	29 (27.6%)	
Hypothyroidism	15 (14.3%)	
Diarrhea	10 (9.5%)	
Neutropenia	9 (8.6%)	5 (4.8%)
Thrombocytopenia	7 (6.7%)	3 (2.9%)
Vomiting	6 (5.7%)	
Pneumonia	4 (3.8%)	2 (1.9%)
Peripheral neuropathy	3 (2.9%)	
Pyrexia	3 (2.9%)	1 (1.0%)
Venous thrombosis	3 (2.9%)	1 (1.0%)
Dizziness	2 (1.9%)	
Cough	2 (1.9%)	

### PFS and OS

**Figure 1:**
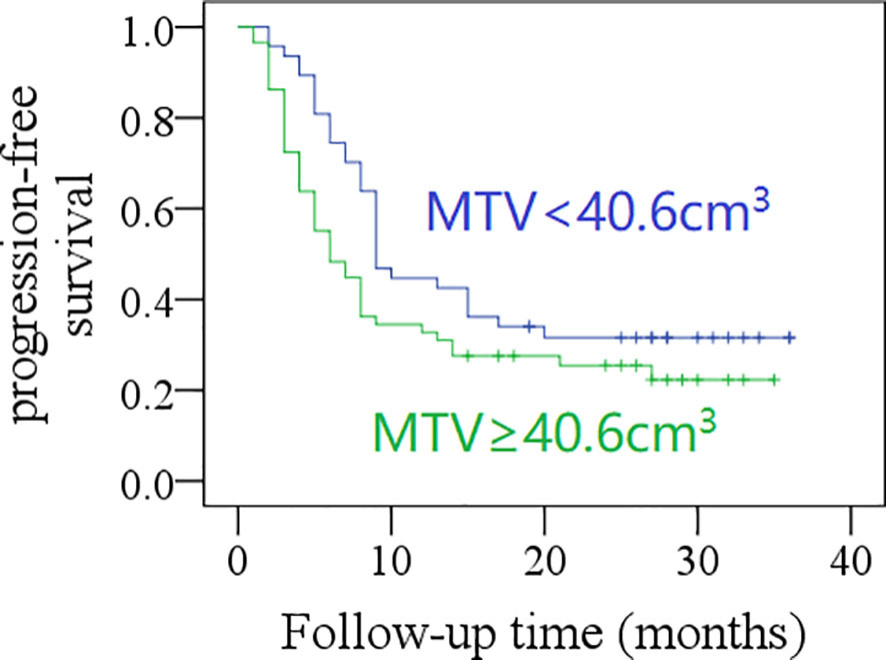
Comparison of progression-free survival in patients with different total metabolic tumor volumes (MTV) (*p* = 0.057).

**Figure 2:**
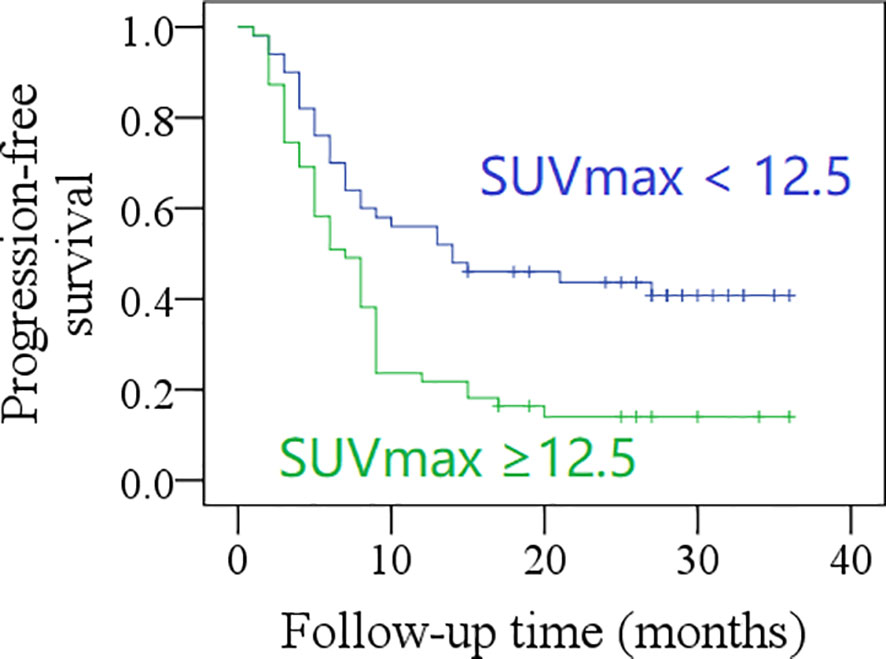
Comparison of progression-free survival in patients with different total maximum standard uptake values (SUVmax) (*p* = 0.001).

After a mean follow-up time of 20.3 months (range: 2–36), the 2-year PFS rate was 31% in patients with total MTV <40.6 cm^3^, and 21% in those with total MTV ≥40.6 cm^3^; the difference was not significant (*p* = 0.057, **Figure 1**). The 2-year PFS rate was 39% in patients with total SUVmax <12.5, and 14% in those with total SUVmax ≥12.5, the difference was significant (*p* = 0.001, **Figure 2**). Further Cox model confirmed the independence of total SUVmax in decreasing the PFS (**Table 5**).

**Table 5:** Univariate analysis and Cox model for progression-free survival (PFS) and overall survival (OS).

Variables	PFS			OS		
	Univariate	Cox model		Univariate	Cox model	
	*p*-Value	*p*-Value	HR [95% CI]	*p*-Value	*p*-Value	HR [95%CI]
Age	0.367			0.631		
Sex	0.167			0.432		
ECOG	0.641			0.048		
Smoker	0.222			0.254		
Tumor site	0.107			0.189		
p16	0.049	0.023	0.82 [0.67–0.98]	0.667		
Total SUVmax	0.001	0.014	2.76 [1.25–5.89]	0.071		
Total MTV	0.057			0.017	0.018	3.86 [1.35–7.88]
CPS				0.099		
<1						
1–20		0.087	0.88 [0.42–1.05]			
≥20	0.002	0.004	0.68 [0.37–0.83]			
Chemotherapy	0.035	0.034	0.86 [0.67–0.99]	0.032	0.077	0.98 [0.46–1.28]

ECOG, Eastern Cooperative Oncology Group score; Total SUVmax, total max standard uptake value; Total MTV, total metabolic tumor volume; CPS, combined positive score.

**Figure 3:**
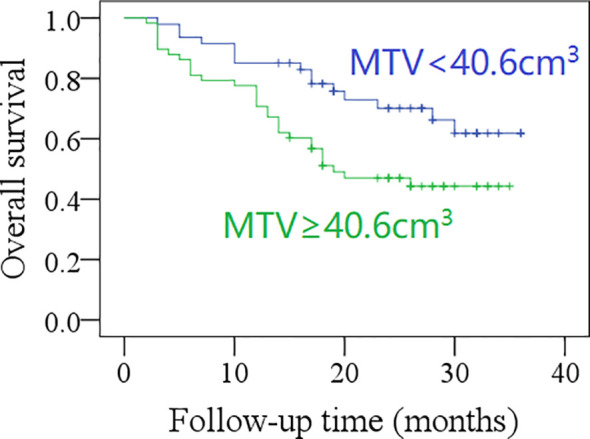
Comparison of overall survival in patients with different total metabolic tumor volumes (MTV) (*p* = 0.017).

**Figure 4:**
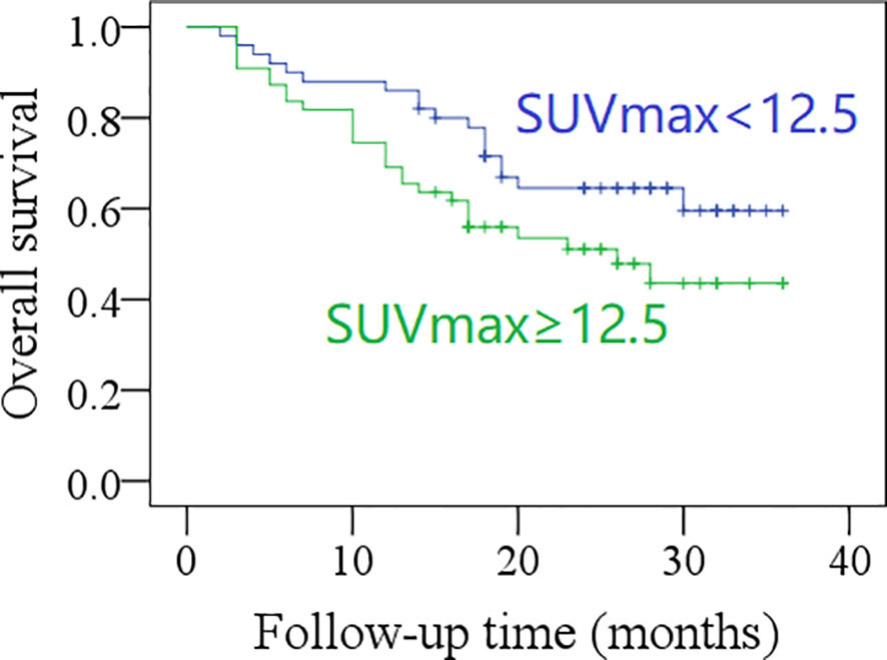
Comparison of overall survival in patients with different total maximum standard uptake values (SUVmax) (*p* = 0.071).

The 2-year OS rate was 62% in patients with total MTV <40.6 cm^3^, and 42% in those with total MTV ≥40.6 cm^3^, the difference was significant (*p* = 0.017, **Figure 3**). The 2-year OS rate was 60% in patients with total SUVmax <12.5, and 42% in those with total SUVmax ≥12.5; the difference was not significant (*p* = 0.071, **Figure 4**). Further Cox model confirmed the independence of total MTV in decreasing the OS (**Table 5**).

## Discussion

The most important finding in this study is that total MTV was associated with the efficacy of PD-1 inhibitor in unresectable HNSCC, but this could not translate into better PFS. Furthermore, total SUVmax had little effect on treatment response to immunotherapy, but total SUVmax ≥12.5 was related to worse PFS.

Several researchers had aimed to explore biomarkers that will predict the efficacy of immunotherapy including PD-L1 expression, tumor mutation burden (15), microbiome (9), and neutrophil/lymphocyte ratio (NLR) (16). Ferris et al. (17) analyzed the OS rates in 240 patients receiving nivolumab with different PD-L1 expression levels and found that the two groups (<1% *vs*. ≥1%) had similar estimated OS rates at 18, 24, and 30 months. However, in a study by Burtness et al. (3), patients with CPS >20 had the highest OS rate after treatment with pembrolizumab alone, and patients with CPS ranging from 1 to 20 had superior OS after being treated with chemotherapy and pembrolizumab rather than pembrolizumab alone. Additionally, Rizvi et al. (15) found that improved objective response, durable clinical benefit, and progression-free survival were noted in tumors with higher nonsynonymous mutation burden in nonsmall cell lung cancer. Routy et al. (9) presented in animal models that primary resistance to immunotherapy was attributable to abnormal gut microbiome composition, and that oral supplementation with *Akkermansia muciniphila* after fecal microbiota transplantation with nonresponder feces restored the efficacy of PD-1 blockade. However, both of them were not applicable in clinical practice. Some authors have assessed the role of blood cell count in immunotherapy, such as NLR; Park et al. (16) found that low NLR at week 6 of therapy and decreased NLR during treatment were both associated with a longer PFS in advanced HNSCC, but as previously stated, the NLR was a nonspecific index, easily affected by local or system inflammation (18). Therefore, continuous exploration for a reliable predictive biomarker for immunotherapy is required.

MTV was a commonly analyzed parameter of PET-CT; it referred to the volume of tumor tissue with high metabolic activity. Our previous studies have confirmed its negative effect on disease prognosis in HNSCC (11), but whether it is associated with the efficacy of immunotherapy in HNSCC remains unknown. We were the first to find that tumors with greater total MTV have better ORR than those with smaller total MTV, and that the improved treatment response contributed to similar PFS rates in the two groups; the finding was very interesting, on the one hand, usually the MTV reflected the tumor burden, it took length diameter and depth of invasion into consideration. Sridharan et al. (8) introduced a different calculation method, which was defined as the sum of the largest diameter of all measurable lesions; the median tumor burden was 5.4 cm and had no relationship with clinical pathologic variables, and tumor burden >5.4 cm was inversely correlated with clinical benefit of immunotherapy and OS. However, in a study by Suzuki et al. (19), which used the same concept of tumor burden, the authors did not report the aforementioned association. These inconsistencies in the reports might have uncovered the inferiority of tumor burden calculation using tumor diameter. On the other hand, the MTV also reflected the functional and biologic status of the tumor due to the biologic features of glucose. High total MTV means a more advanced HNSCC, which is likely to have PD-L1 overexpression (20). Negative prognostic effect of MTV on surgically treated HNSCC has been extensively reported (11), with greater MTV usually related to increased possibility of lymph node metastasis, higher tumor stage, worse disease control and poor prognosis; our study noted after immunotherapy that there was no significant difference regarding PFS between patients with different total MTVs. This indicates the reliability of total MTV in predicting immunotherapy efficacy. However, in a report by Seban et al. (21) who enrolled 63 patients with advanced nonsmall cell lung cancer, high total MTV (>83 cm^3^) was likely to decrease the treatment response rate. As for metastatic melanoma treated with anti-PD-1 therapy, Nakamoto et al. (22) did not comment on whether the clinical benefit of immunotherapy was affected by MTV. These conflicting results might be mostly due to the differences in research objects.

SUVmax was another important parameter of PET-CT, which refers to the ability of tumor tissues to take up tracers; in general, the higher the SUVmax, the higher the possibility and degree of malignancy. A number of researchers have described that patients with high SUVmax were more likely to have larger tumors, advanced stage disease, and poor prognosis (10, 23, 24). Whether or not SUVmax can be used for guiding immunotherapy was never analyzed in patients with HNSCC before, although some researchers have assessed its predictive value in lung cancer (21) and melanoma (22). Both studies concluded that there was no association between SUVmax and clinical response, and we would also like to confirm this negative relationship in HNSCC.

Limitations in this study must be acknowledged: first, its retrospective nature had inherent bias; second, our sample size was small, which decreased our statistic power; third, our follow-up time was limited, there might be more interesting findings in the future; fourth, PD-1 inhibitors consisted of two different drugs in this study, both of which had been confirmed to have good ability of binding to receptor PD-L1 (3, 25).

In summary, in HNSCC, total MTV of ≥40.6 cm^3^ was related to improved clinical response but did not translate into better PFS; total SUVmax had no effect on clinical response but total SUVmax ≥12.5 was associated with worse PFS.

## Data Availability Statement

The original contributions presented in the study are included in the article/Supplementary Material. Further inquiries can be directed to the corresponding author.

## Ethics Statement

Henan Cancer hospital institutional research committee approved our study, and all participants signed an informed consent agreement. The patients/participants provided their written informed consent to participate in this study.

## Author Contributions

All authors have contributed to the concept design, data collection, data analysis, manuscript writing, and manuscript revision. All authors contributed to the article and approved the submitted version.

## Conflict of Interest

The authors declare that the research was conducted in the absence of any commercial or financial relationships that could be construed as a potential conflict of interest.

## Publisher’s Note

All claims expressed in this article are solely those of the authors and do not necessarily represent those of their affiliated organizations, or those of the publisher, the editors and the reviewers. Any product that may be evaluated in this article, or claim that may be made by its manufacturer, is not guaranteed or endorsed by the publisher.
